# Modeling the potential distribution of *Hippophae rhamnoides* in China under current and future climate scenarios using the biomod2 model

**DOI:** 10.3389/fpls.2025.1533251

**Published:** 2025-04-04

**Authors:** Tingjiang Gan, Zhipeng He, Danping Xu, Juan Chen, Honghua Zhang, Xinju Wei, Zhihang Zhuo

**Affiliations:** ^1^ Engineering Research Center of Chuanxibei Rural Human Settlement (RHS) Construction, Mianyang Teachers’ College, Mianyang, China; ^2^ College of Life Science, China West Normal University, Nanchong, China; ^3^ College of Architecture, Changsha University of Science and Technology, Changsha, China

**Keywords:** *H. rhamnoides*, biomod2, climate change, potential distribution, environmental variable

## Abstract

**Introduction:**

Hippophae rhamnoides, a temperate species with a transcontinental distribution spanning Eurasia, demonstrates preferential establishment in water-limited ecosystems (arid/semi-arid zones), particularly occupying high-elevation niches with skeletal soils and high solar flux. This ecologically significant plant, prized for dual ecological provisioning and economic services, shows biogeographic concentration in China’s northern desertification belts, northwestern Loess Plateau, and southwestern montane corridors. Studying the possible areas where H. rhamnoides may be found can offer a scientific foundation for the protection and sustainable management of its resources.

**Methods:**

This study utilized the biomod2 software to assess an integrated model based on 312 distribution points and 23 environmental factors. Furthermore, a modeling analysis was conducted to examine how the geographical distribution of H. rhamnoides changes over time under the SSP245 scenario.

**Results:**

The findings show that the distribution of H. rhamnoides is primarily affected by three factors: annual mean temperature, temperature seasonality and mean temperature of the coldest quarter. Currently, H. rhamnoides is predominantly distributed in the provinces of Shanxi, Shaanxi, Gansu, Hebei, Yunnan, Xinjiang, Tibet, Sichuan, Qinghai, and Ningxia. The suitable habitat covers an area of 212.89×10⁴ km², which represents 22.15% of China’s total land area. Within this region, high, medium, and low suitability areas make up 23.15%, 22.66%, and 54.20% of the suitable habitat, respectively.

**Discussion:**

In the future, the centroid of the suitable habitat for H. rhamnoides is expected to gradually shift northwest, with a trend of increasing suitability in the west and decreasing suitability in the east. This study aims to provide an in-depth exploration of the distribution of H. rhamnoides and the influence of environmental factors on it from a geographical perspective. These results are important for improving the conservation, management, cultivation, and propagation of H. rhamnoides, while also offering a scientific foundation for the research of other valuable plant species.

## 1 Introduction

Sea buckthorn (*Hippophae rhamnoides L.*) is a plant belonging to the Elaeagnaceae family and the Hippophae genus, originally native to Europe and Asia (Xia et al., 2023). *Hippophae rhamnoides* is one of the oldest plants in the world, originating approximately 65 million years ago during the Cretaceous period. This species has the highest natural distribution and cultivation density in China, where it is rich in natural resources (Lv et al., 2021). It contains a variety of compounds, including rich nutritional components such as proteins, minerals, and vitamins, as well as various bioactive substances, including flavonoids, terpenoids, steroids, organic acids, and alkaloids (Ma et al., 2023). In China, this species is primarily distributed in north, northwest, and southwest (Hu et al., 2024). As a valuable biological resource with significant potential, *H. rhamnoides* has extensive applications in land restoration, soil erosion prevention, and energy sources (Mihal et al., 2023). And it was once listed as an endangered species in the IUCN Red List of Threatened Species in 2017 (IUCN, 2017). Currently, there is a relatively substantial amount of research of *H. rhamnoides*, with existing studies primarily focusing on fields such as traditional Chinese medicine, chemistry, and biology (Kukina et al., 2020; Balke et al., 2022; Lee et al., 2023). However, research on the potential distribution areas of *H. rhamnoides* remains limited. Therefore, understanding its distribution and habitat preferences is crucial for effective conservation efforts.

Research on the effects of climate change on the geographical distribution of plants has increasingly focused on species distribution models (SDMs), which are grounded in ecological niche theory. These models utilize specific algorithms to analyze known plant distributions and their corresponding environmental factors, thereby predicting suitable areas for plant species (Kearney et al., 2010; Zhang et al., 2019). Species distribution models are commonly employed to forecast potential distributions across different scales. These include global patterns of species diversity, the movement of invasive species at regional levels, and the modeling of local population distributions (Fu et al., 2024; Wang et al., 2024). Current, widely used species distribution models include Generalized Linear Models (GLMs), Genetic Algorithm for Rule-set Production (GARP), Maximum Entropy (MaxEnt), and the Bioclimatic Model (BIOCLIM) (Gao et al., 2024). Typically, these models combine species distribution data with climate factors to clarify shifts in distribution and pinpoint the key environmental factors that shape species distribution patterns (Wu et al., 2024). Due to differences in their underlying principles and algorithms, each model has unique advantages and limitations. However, the performance of these models may also become unstable when input data changes (Grimmett et al., 2020). As a result, combining predictions from several models into an ensemble approach can greatly improve the accuracy of forecasts (Jinga et al., 2021). Therefore, the Biomod2 modeling platform, which combines multiple models, was developed in 2003 and has since been widely recognized in this field (Thuiller et al., 2009; Bi et al., 2013). The platform integrates various modeling algorithms, allowing users to choose different models and customize them by modifying initial conditions, model types, parameters, and boundary settings to optimize predictive results.

Climate change has become a major driver of changes in the distribution of plant species across the globe. Understanding how climate change impacts the species’ distribution is crucial for its conservation. In China, significant climate shifts have occurred over the last century, with an average temperature increase of approximately 1.5°C and changes in precipitation patterns, such as more frequent droughts in northern regions (Zhou et al., 2020). These shifts are expected to influence the distribution of *H. rhamnoides*, potentially leading to range contractions in southern areas and northward expansions (Qiu et al., 2025). The species’ ecological affinities, such as its tolerance to cold temperatures and drought conditions, make it an ideal subject for studying adaptation to climate change (Cheng et al., 2024).

The objective of this research is to assess the spatiotemporal shifts in the potential distribution range of *H. rhamnoides*, identify climate change area in future scenarios, and pinpoint key factors that could affect changes in its geographical range. Understanding these shifts is crucial because climate change is expected to significantly impact the distribution of many plant species, including *H. rhamnoides*. By identifying regions where its habitat may be most vulnerable or expanding, this research can guide targeted conservation efforts, ensure the sustainable management of *H. rhamnoides* in China, and contribute to broader biodiversity preservation strategies in the face of global climate changes.

## 2 Materials and methods

### 2.1 Sample data acquisition and screening

**Figure 1:**
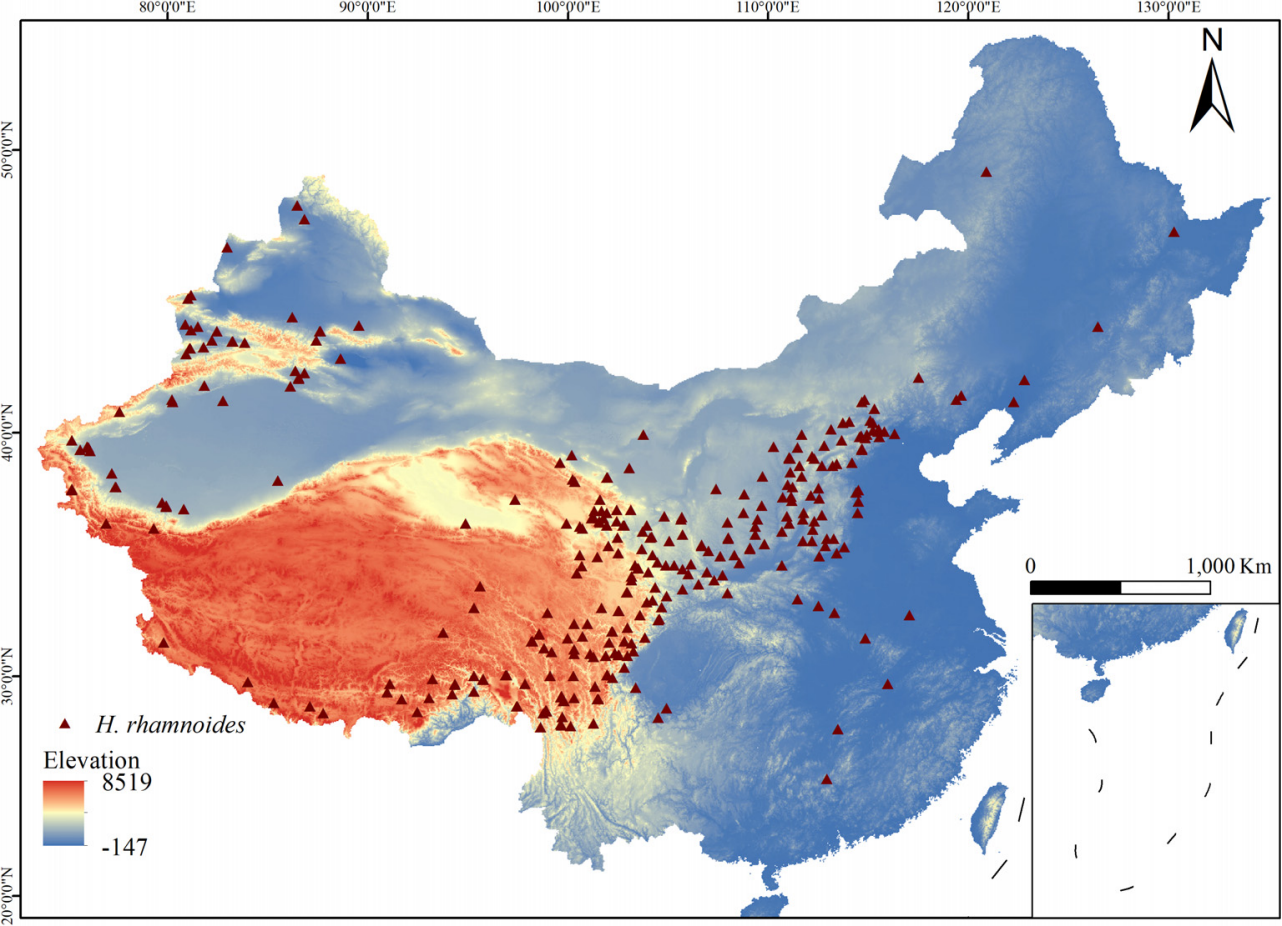
Geographic distribution records of *H. rhamnoides*. (The color bar indicates area Elevation, Brown triangles: *H. rhamnoides* distribution points.).

The occurrence data for *H. rhamnoides* used in this study were obtained from several sources, including the Global Biodiversity Information Facility (GBIF, http://www.gbif.org/), the Chinese Natural Plant Specimen Museum (http://www.cfh.ac.cn), the Chinese Digital Herbarium (http://www.cvh), and relevant field surveys, resulting in a total of 1,384 occurrence records. These records were filtered to remove non-natural occurrences and duplicate data. To reduce potential clustering bias, only one occurrence point was kept for each 2.5 km grid (5 km × 5 km). Ultimately, 312 valid samples were selected, as shown in **Figure 1**.

### 2.2 Collection and evaluation of environmental data

In predicting species distribution, environmental factors play a crucial role. This study selected four types of influencing factors. First, 19 climatic factors were selected from the WorldClim 2.1 climate database (https://worldclim.org/) (Petrie et al., 2021). Second, considering the ecological significance of soil factors and the characteristics of *H. rhamnoides*, 11 soil factors were chosen from the Harmonized World Soil Database (HWSD) provided by the Food and Agriculture Organization (FAO) (http://www.fao.org/faostat/en/). Third, three topographical factors were obtained from the National Centers for Environmental Information (NCEI) of the National Oceanic and Atmospheric Administration (NOAA) (https://www.ngdc.noaa.gov/). Finally, the study acquired Category IV environmental covariates potentially influencing X distribution, comprising: land cover classification and fractional vegetation cover from Global Maps (https://globalmaps.jp/); Human Footprint Index and Human Impact Index sourced from the Center for International Earth Science Information Network (CIESIN)(http://sedac.ciesin.columbia.edu/wildareas/); and mean annual erythemal UV dose derived from the Helmholtz Centre for Environmental Research - UFZ databases(https://www.ufz.de/gluv/index.php?en=32435). For future climate projections, this study used bioclimatic factors from the medium-emission scenario of the BCC-CSM2-MR climate model, developed by the Beijing Climate Center (BCC), under the SSP245 scenario. The data were analyzed for three time periods: 2041–2060, 2061–2080, and 2081–2100. Due to the strong autocorrelation among environmental factors and the fact that not all factors are essential for predicting potential distributions, this study employed R software to conduct separate correlation analyses of bioclimatic factors and soil factors. The analysis results were used to assess the multicollinearity among environmental factors.

### 2.3 Building and evaluation of the model

Given the limitations of individual models, utilizing an ensemble approach for predictions is expected to produce more reliable results (Wang et al., 2023). This study employed eight distinct model types to predict the current and future habitat suitability for *H. rhamnoides*. The models used include two regression techniques (Generalized Linear Model (GLM) and Multivariate Adaptive Regression Splines (MARS)), four machine learning methods (Artificial Neural Network (ANN), Random Forest (RF), Generalized Boosted Model (GBM), and Classification Tree Analysis (CTA)), one classification model (Flexible Discriminant Analysis (FDA)), and one range envelope approach (Surface Range Envelope (SRE)).

Biomod2 can be used to develop integrated models that require both species distribution and pseudo-absence data. The software provides various methods for generating pseudo-absence points based on background data. In this study, 1,000 pseudo-absence points were randomly generated using the ‘random’ command for model simulation (Wen et al., 2022). The ‘biomod tuning’ command was applied to optimize model parameters, with 75% of the sample data used for training and the remaining 25% reserved for validation. Equal weights were assigned to both distribution and pseudo-absence data, and this procedure was repeated 10 times. For model simulations, a weighted averaging method was employed, retaining only models with a TSS ≥ 0.75 to construct the ensemble model. Predictive accuracy was evaluated using the Receiver Operating Characteristic (ROC) curve and the True Skill Statistic (TSS), and the robustness of the model results was further calibrated and validated (Xian et al., 2023). The output range for the ROC and TSS metrics is from 0 to 1.0.Values between 0.85 and 1.0 indicate excellent predictive performance, 0.7 to 0.85 indicates good performance, 0.5 to 0.7 reflects fair performance, and 0.4 to 0.55 suggests moderate performance. Scores below 0.4 indicate poor predictions, with areas below this threshold considered unsuitable. Based on these established thresholds, suitable areas can be further categorized into low, medium, and high suitability zones.

### 2.4 Classification and centroid change of potentially suitable distributions

The results of the Biomod2 model were evaluated using TSS and ROC scoring to select the appropriate ensemble model. Subsequently, the suitable areas were classified using the Jenks Natural Break Classification (NBC) method (Zhao R, et al., 2024). After establishing the ensemble model, it was projected onto future environmental factors to predict potential geographic distribution. The resulting distribution maps were then normalized using ArcGIS 10.8. Based on the results from Biomod2, areas above the threshold were designated as suitable zones. The top 10% of these suitable areas were classified as high suitability zones, while the remaining suitable areas were divided into medium and low suitability zones. Finally, the raster calculator was used to reclassify and allocate data under different climatic conditions.

Additionally, the raster calculation function in ArcGIS was utilized to compute the mean values, thereby determining the centroid of suitable distribution areas for *H. rhamnoides*. This analysis reflects the geographical migration process of the target species. The SDMTool toolbox was used to calculate the migration distances of the centroids of suitable areas for *H. rhamnoides* across different time periods. The analysis focused on the temporal and spatial changes in high-suitability habitats during the 1950s, 1970s, and 1990s, as well as the associated shifts in centroids.

## 3 Results

### 3.1 Model accuracy and key factors

**Figure 2:**
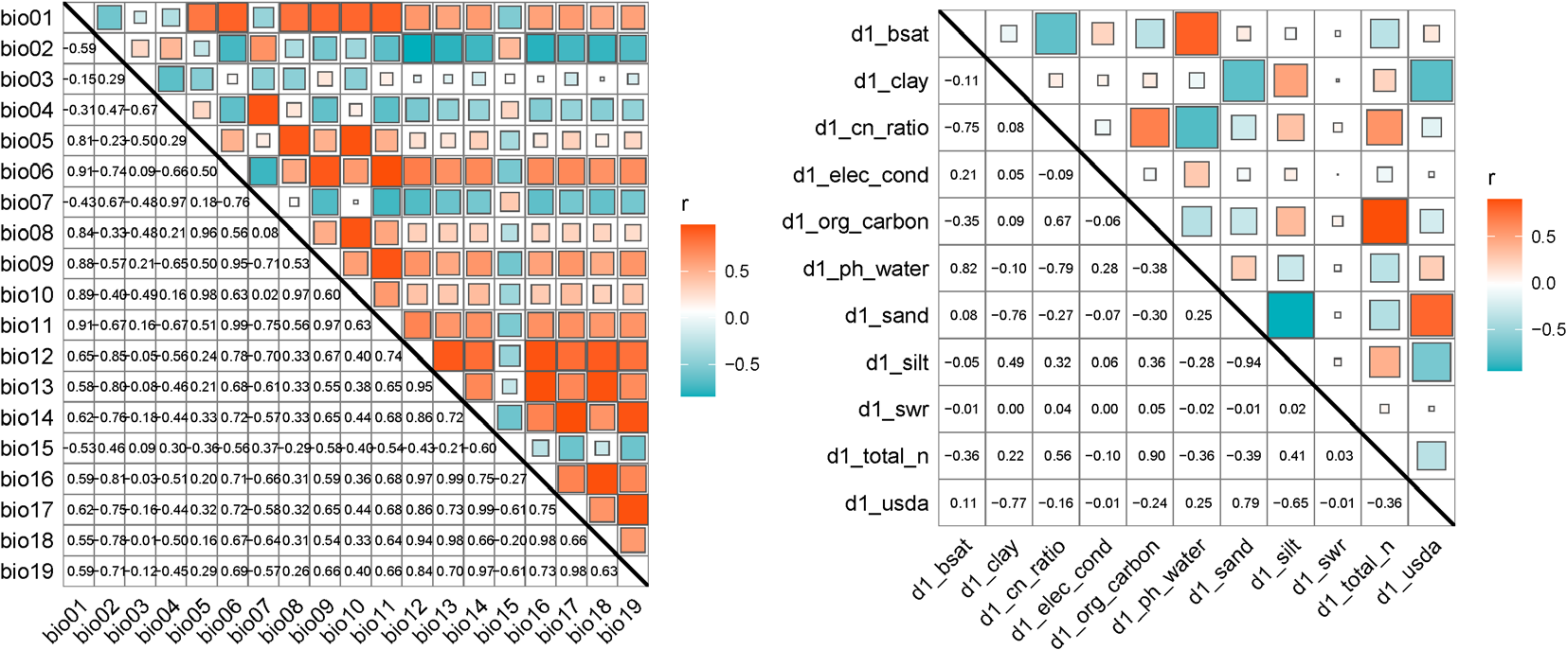
Correlation coefficient matrix of 19 bioclimatic factors and 11 soil factors. (Red indicates a positive correlation, blue indicates a negative correlation, and the strength of Pearson’s correlation coefficient (r) increases as the square of the correlation grows larger.).

Bioclimatic and soil factor correlation analysis is shown in Figure (**Figure 2**), removing environmental factors with a correlation greater than 0.8 to reduce the risk of model overfitting due to multicollinearity. Considering the biological characteristics of *H. rhamnoides*, the final selection retained the ecologically most important environmental factors, resulting in a total of 23 factors (**Table 1**) being included in the model construction for *H. rhamnoides*.

**Table 1:** 23 variables of model construction.

Abbreviation	Description
bio01	Annual mean temperature
bio03	Isothermally
bio04	Temperature seasonality
bio05	Max temperature of the warmest month
bio06	Min temperature of the coldest month
bio09	Mean temperature of the driest quarter
bio11	Mean temperature of the coldest quarter
bio17	Precipitation of the driest quarter
aspect	The direction or orientation of the earth’s surface
elev	Elevation of theterrain
slope	Slope or obliquity of the terrain
d1_clay	clay content
d1_cn_ratio	Carbon nitrogen ratio
d1_elec_cond	Electric conductivity
d1_ph_water	PH
d1_sand	Sand content
d1_swr	Soil water regime
d1_total_n	Total nitrogen content
d1_usda	Topsoil texture classification
gm_lc_v3	Land cover type
gm_ve_v2	Percentage of vegetation coverage
hf_v2geo1	Human footprint and human impact index
annual_mean uv-b	Average annual UV exposure

**Figure 3:**
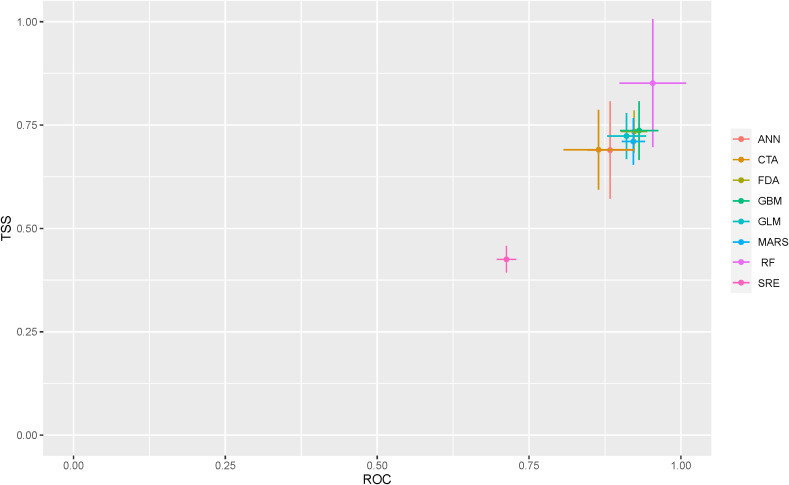
TSS and ROC evaluations of each single model of *H. rhamnoides*.

The results show that all 8 models were successfully run. The ‘biomod_tuning’ function was used to optimize model parameters, and these parameters were evaluated in each iteration using the selected methods (ROC, Kappa, or TSS). The optimized parameters produced the most accurate predictive results. Overall, the findings indicate that the RF model is the most effective for predicting the potential spatial distribution of *H. rhamnoides*. In comparison, the GBM, FD, CTA, ANN, and GLM models scored slightly lower, with the RF model achieving an average Kappa coefficient of 0.87 and an average TSS of 0.91 (**Figure 3**). The models with the poorest performance were MARS and SRE, which failed to meet the accuracy criteria. The remaining five models showed moderate performance levels. Of the eight models that passed the accuracy tests, six were selected to construct the ensemble model, which achieved a Kappa coefficient of 0.703, a TSS of 0.830, and an AUC of 0.97.

**Figure 4:**
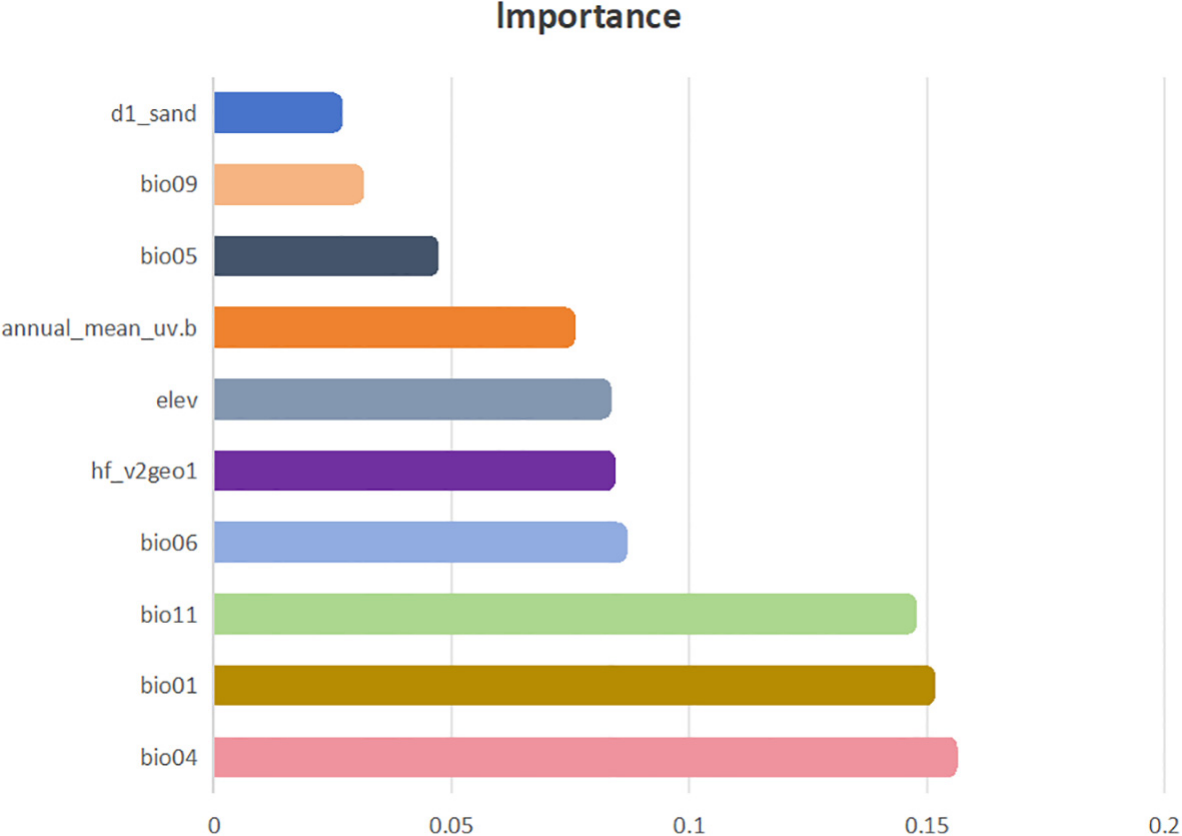
Percentage contribution of top ten environmental factors to the final model of *H. rhamnoides*.

**Figure 5:**
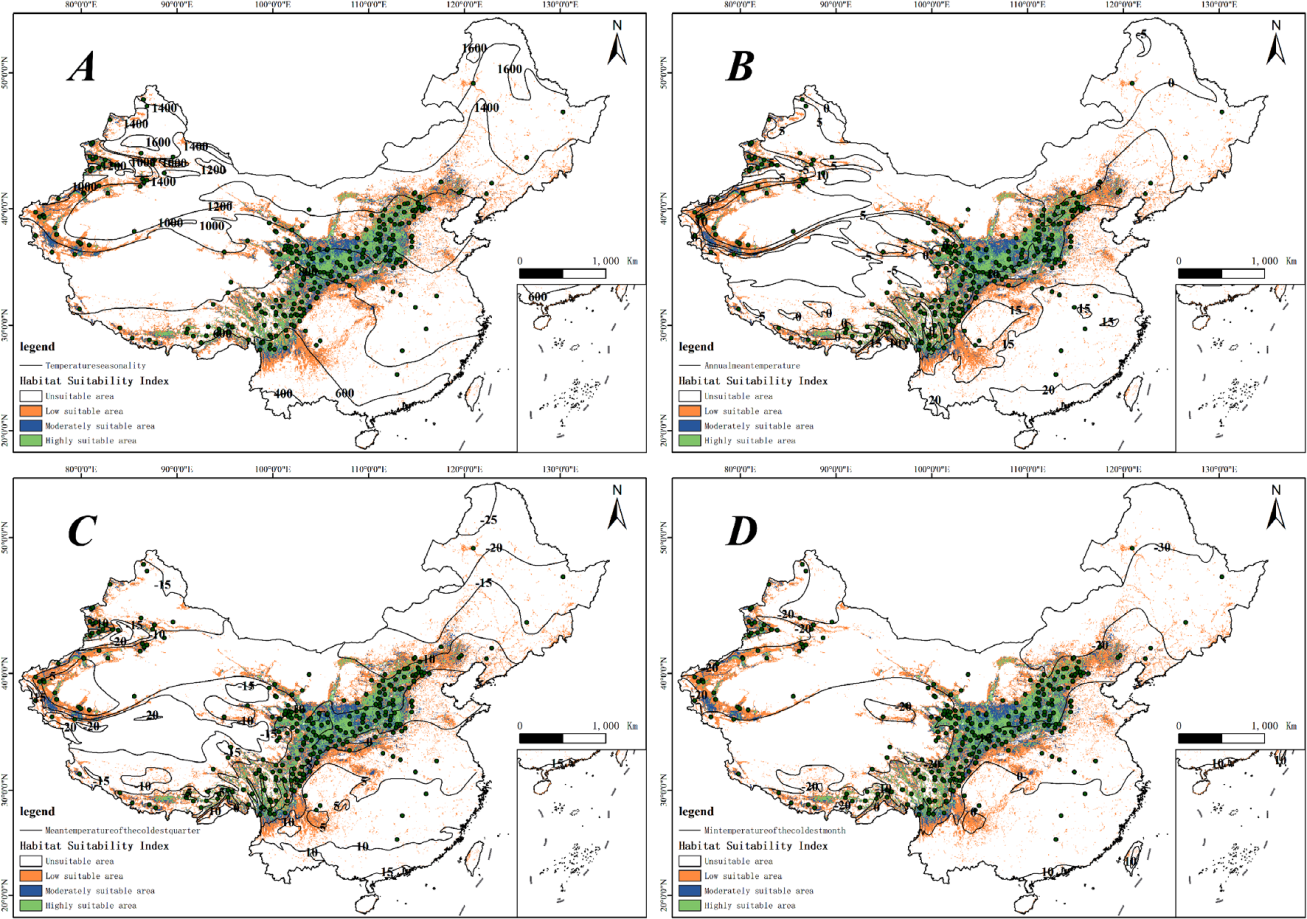
Composite graph displaying the distribution of *H. rhamnoides* points, habitat suitability, and contour lines for environmental factors. **(A)** Temperature seasonality (bio04), **(B)** Annual mean temperature (bio01), **(C)** Mean temperature of the coldest quarter (bio11), **(D)** Minimum temperature of the coldest month (bio06).

Through the use of the Biomod2 platform, we evaluated how each environmental factor contributed to the modeling process. Our analysis revealed that various models employed different combinations of factors, with the influence of each factor on the models differing considerably. Using the best-performing combined model, we examined the significance of environmental factors influencing the distribution of *H. rhamnoides* (**Figure 4**). The analysis results indicate that bioclimatic factors have a greater impact on predicting the distribution of *H. rhamnoides* compared to other factors. This is followed by the human footprint and human activity index, with terrain factors, UV radiation, and soil factors having progressively lesser impacts. The most influential bioclimatic factors in the model for *H. rhamnoides* included seasonal temperature (bio04, 15.43%), average temperature (bio01, 14.96%), the average temperature during the coldest quarter (bio11, 14.58%), and the minimum temperature of the coldest month (bio06, 8.48%). These four bioclimatic variables made up a significant portion of the total contribution, with a cumulative contribution rate of 53.44%. In this study, the four environmental factors were visualized alongside the current potential distribution (**Figure 5**). and it was found that the distribution point data of *H. rhamoides* appeared within the optimal threshold of various environmental factors, indicating that the Biomod2 comprehensive model had good performance. Additionally, among the remaining 19 factors used in model construction, only three—human footprint and human activity index (hf_v2geo1, 8.24%), elevation (elev, 8.15%), and annual average UV radiation (annual_mean_uv.b, 7.39%)—had contribution rates exceeding 5%.

### 3.2 The distribution pattern of *H. rhamnoides* under current conditions

**Figure 6:**
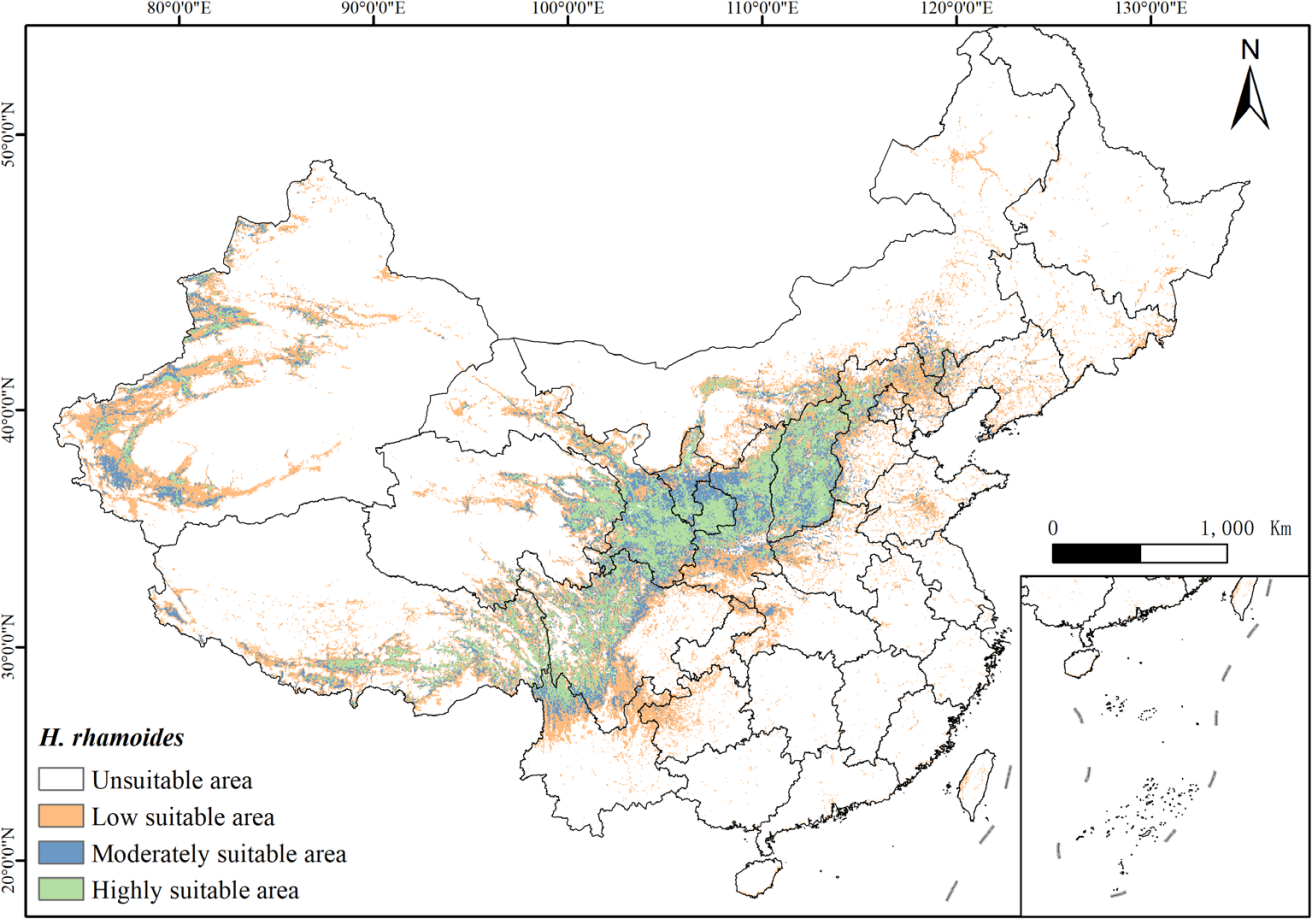
The distribution of suitable habitats of *H. rhamnoides* varies across different regions of China under the current climate conditions (green: high suitability area; blue: moderately suitable area; yellow: low suitable area; white: unsuitable area).

Under current climatic conditions, the potential distribution of *H. rhamnoides* is illustrated in **Figure 6**. The area of highly suitable habitat is 492,700 km², while moderately suitable habitat covers 482,300 km², and low suitability habitat spans 1,153,800 km². These areas account for 23.15%, 22.66%, and 54.20% of the total suitable habitat, respectively. Overall, suitable habitat represents 22.15% of China’s total land area. The suitable areas are primarily concentrated in regions such as Sichuan, Gansu, Qinghai, Ningxia, Shaanxi, and Shanxi, with smaller distributions also found in Yunnan, Xinjiang, and Tibet (**Figure 6**). Notably, suitable habitats for *H. rhamnoides* are mainly located near the Yellow River basin.

Combining the climate data reveals a significant overlap between the habitat suitability for *H. rhamnoides* and temperature levels. Under excessively low-temperature conditions, the plant is unsuitable for cultivation and growth.

### 3.3 Changes in the habitat areas of *H. rhamnoides* under future conditions

**Figure 7:**
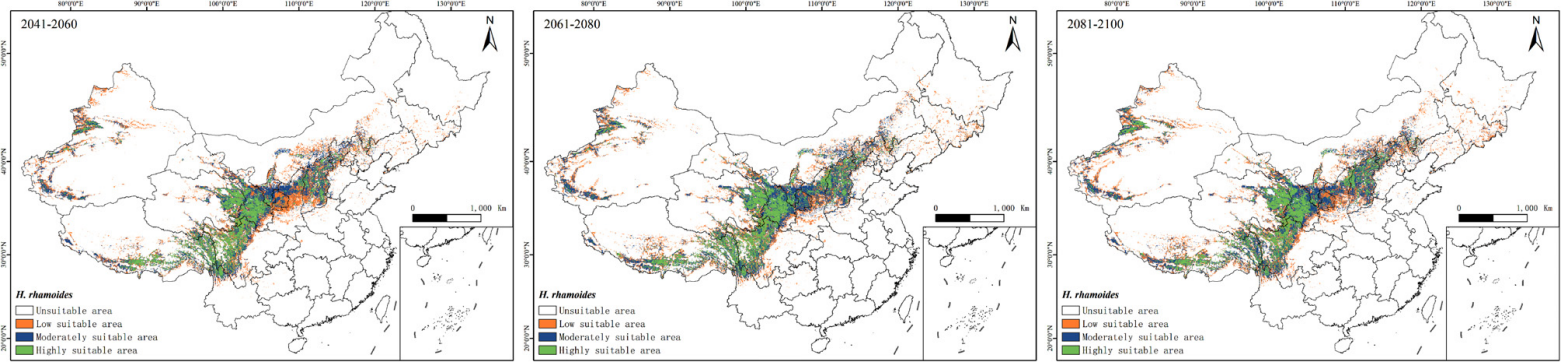
Prediction map of the potentially suitable area of *H. rhamnoides* in China at different periods under the SSP245 climate scenario (green: high suitability area; blue: moderately suitable area; yellow: low suitable area; white: unsuitable area).

The prediction results indicate that under the SSP245 scenario, the future potential geographic distribution of *H. rhamnoides* will be similar to its current potential distribution (**Figure 7**). However, there is a gradual trend of contraction in the suitable habitat area. By the 2050s, the suitable area for *H. rhamnoides* is projected to be approximately 1,372,600 km², representing a decrease of 35.53% compared to the current area. Within this suitable habitat, the proportions of high, moderate, and low suitability areas are expected to be 32.98%, 25.19%, and 41.83%, respectively. By the 2070s, the suitable area for *H. rhamnoides* is projected to be approximately 1,477,400 km², reflecting a decrease of 30.60% compared to the current area. Within this suitable habitat, the proportions of high, moderate, and low suitability areas are expected to be 34.13%, 36.60%, and 29.27%, respectively. By the 2090s, the suitable area for *H. rhamnoides* is projected to be approximately 1,521,300 km², representing a decrease of 28.54% compared to the current area. The proportions of suitable habitats are expected to be 35.70% for high suitability, 37.36% for moderate suitability, and 26.94% for low suitability. Overall, the areas of high, moderate, and low suitability show a trend of initial reduction followed by gradual expansion.

### 3.4 Changes in the centroid of suitable areas for *H. rhamnoides* in the future

**Figure 8:**
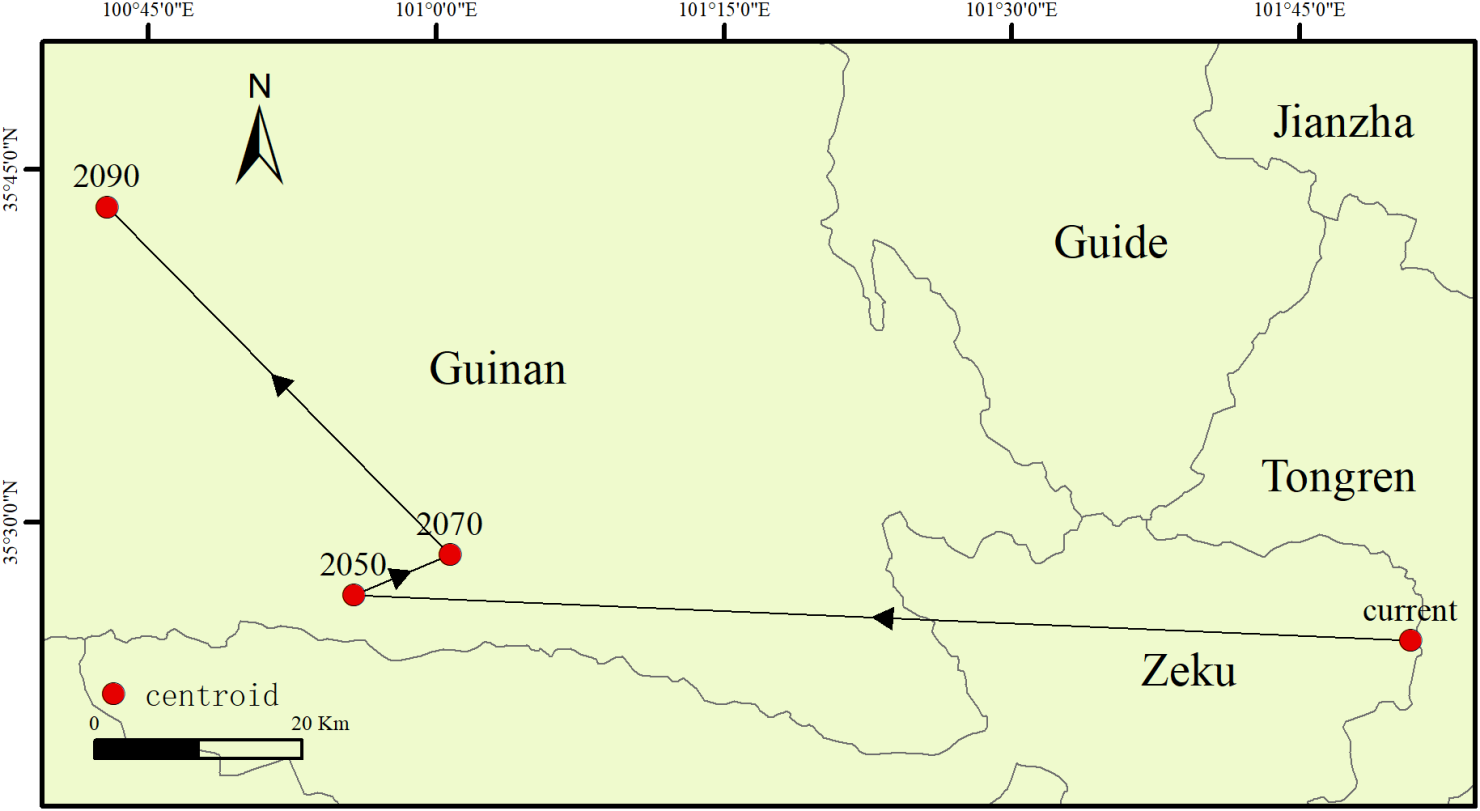
The change in the centroid of the potential distribution area of *H. rhamnoides* in China.

As shown in **Figure 8**, the centroid location and movement direction of the suitable area for *H. rhamnoides* were calculated using ArcGIS. Currently, the centroid is located in Zeku County, Qinghai Province, at coordinates 102.478° E, 35.263° N. By the 2050s, the centroid is projected to move 205.37 km southwest to Guinan County, Qinghai Province, with new coordinates of 100.712° E, 34.825° N. By the 2070s, the centroid is expected to shift 53.39 km northeast, remaining in Guinan County at coordinates 101.129° E, 35.020° N. By the 2090s, it will move 60.71 km northwest but will still be located in Guinan County, at coordinates 100.658° E, 35.246° N.

**Figure 9:**
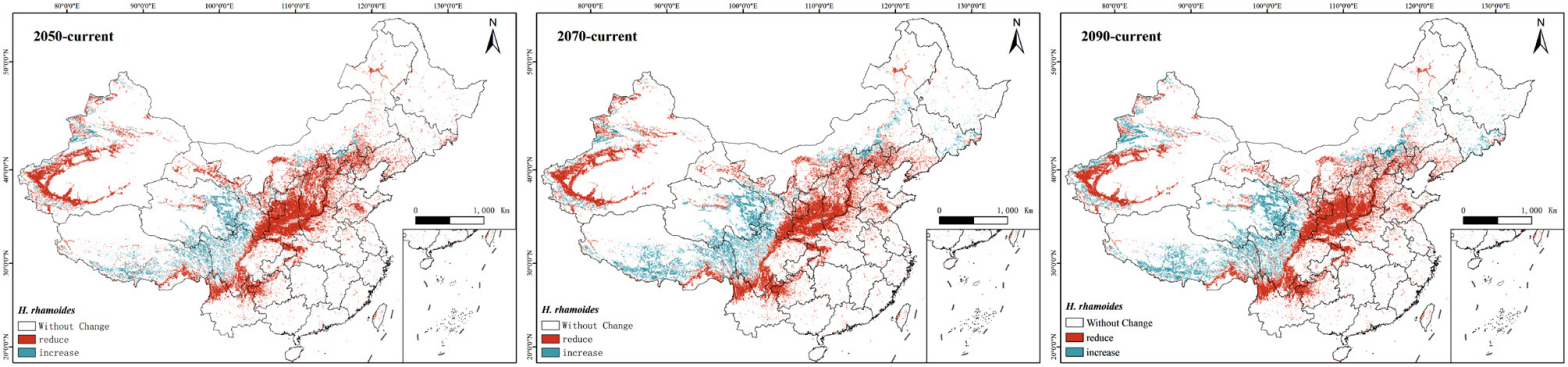
A comparison of changes in the suitable area for *H. rhamnoides* under the SSP245 scenario with the currently suitable area over future periods (red: degradation or loss of suitable areas; blue: the rise of suitable areas).

Additionally, the study utilized ArcGIS to conduct a comparative analysis of suitable areas under the current scenario and those projected for the SSP245 scenario in the 2050s, 2070s, and 2090s (**Figure 9**). The results indicate that in future climate scenarios, regions such as the Hai River Basin and the Yellow River Basin exhibit trends of degradation or loss of suitable areas, while suitability levels in the Southwest Basin are increasing. Over time, this trend becomes increasingly pronounced.

In summary, based on the predicted future climate conditions, the centroid of *H. rhamnoides* distribution is expected to shift slightly westward over time.

## 4 Discussion

The research results show that that the optimal Temperature seasonality for *H. rhamnoides* ranges between 600 and 1200 (**Figure 5A**). Seasonal temperature influences plants in various ways; suitable temperature conditions can promote healthy growth, while extreme temperature fluctuations may result in growth stagnation or the onset of diseases (Albuquerque et al., 2023). *Hippophae rhamnoides* can grow across a broad range of seasonal temperatures, indicating its adaptability to various environments. Furthermore, this study suggests that *H. rhamnoides* can thrive in harsh, low-temperature conditions, with suitable areas primarily found in regions with an average annual temperature of 0 to 15°C (**Figure 5B**). Its cold tolerance, low growth height, well-developed root system, and thick leaves enable *H. rhamnoides* to effectively produce antifreeze proteins, providing a survival advantage in cold environments (Gupta and Deswal, 2012). This also highlights that *H. rhamnoides* possesses strong vitality, allowing it to effectively maintain soil temperature and moisture. Furthermore, *H. rhamnoides* is well-suited for growth under conditions where the minimum temperature in the coldest month ranges from -20 to 10°C (**Figure 5C**) and can thrive in areas where the lowest temperature during the coldest season falls between -12 and 6°C (**Figure 5D**). These results are similar to those of Arora et al., both indicating that *H. rhamnoides* has strong adaptability, as it can still obtain sufficient energy for growth even under harsh environmental conditions (Arora et al., 2024).

Furthermore, the prediction results indicate that the future geographical distribution of this species will exhibit a spatial divergence trend characterized by westward expansion and eastern contraction. This pattern may be associated with heterogeneous responses to regional climatic factors. Under warming conditions, the western regions, due to their higher elevation, experience less pronounced temperature-induced impacts compared to the eastern lowlands. Notably, the western expansion zones are often constrained by topographic barriers or ecological carrying capacity limitations, suggesting that the actual habitable range may be smaller than model predictions (Liu et al., 2024). Additionally, rising temperatures and increasing extreme climate events may shorten plant growth cycles, leading to reduced habitat suitability. This trend has been corroborated by studies in Europe and parts of Asia, where analogous species have adjusted their habitat ranges under climate change pressures (Duyar et al., 2025). For instance, cold-tolerant species such as *Pinus armandii* and *Betula ermanii* have demonstrated latitudinal or altitudinal shifts to adapt to changing climatic conditions (Huang et al., 2023; Cai et al., 2024). To mitigate climate-driven ecological imbalances, we recommend prioritizing the conservation of habitat connectivity for emerging populations in western regions. Concurrently, adaptive management strategies based on niche substitution should be implemented in eastern decline zones to buffer ecosystem risks.

The ensemble model forecasts that *H. rhamnoides* is predominantly found in the middle and lower reaches of the Haihe, Yellow, and Yangtze River basins. Areas with high habitat suitability are mainly concentrated in the middle and lower reaches of the Yellow River basin, along with certain parts of the Yangtze River basin and southern regions. The favorable water resource environment in the Yellow River basin provides optimal growth conditions for the plants, while the rich vegetation and biodiversity in the plains create a supportive ecosystem for their survival (Zhao G, et al., 2024). The southern region is characterized by abundant forests and biodiversity, with a warm and humid climate. These conditions are highly conducive to agricultural development and plant growth (Zheng et al., 2020; Xie et al., 2024). The global cooling since the mid-Miocene may have facilitated the geographic dispersion and northwestern expansion of *H. rhamnoides* (Su et al., 2023). The results indicate that climate warming may be the primary driver of habitat reduction for *H. rhamnoides*. Although this species demonstrates strong adaptability, it is highly sensitive to rising temperatures, which significantly alter its mountainous habitats. Increased temperatures are prompting *H. rhamnoides* to migrate westward. Furthermore, extreme weather events, such as frequent droughts and heavy rainfall, can adversely affect its growth (Wang et al., 2020). The distribution centroid of *H. rhamnoides* is expected to shift slightly northwest, primarily driven by ecological adaptations to climate change (Wang et al., 2018). This study found that, in response to climate change, the suitable habitat for *H. rhamnoides* is exhibiting an overall trend of contraction in the east and expansion in the west. This shift is primarily attributed to global warming, which is causing gradual increases in temperature and precipitation in western regions of China. Temperature conditions are the main climatic factors influencing the distribution of *H. rhamnoides* (Li et al., 1999). Although *H. rhamnoides* benefits from its unique morphological and physiological structures that provide high water retention capacity, it also exhibits strong adaptability to arid, cold, and nutrient-poor environments (Gupta and Deswal, 2012). Furthermore, numerous studies have shown that *H. rhamnoides* requires specific moisture conditions for optimal growth and development. Water stress can significantly impair photosynthesis and adversely affect overall growth and fruit yield. Therefore, maintaining appropriate moisture levels is crucial for its healthy development (Jin et al., 2006). In the distribution areas of *H. rhamnoides*, the growing season is characterized by elevated temperatures and increased transpiration, underscoring the critical importance of both precipitation and temperature during this time (Zhang et al., 2022). The ensemble model analysis further supports that the key factors affecting the distribution of *H. rhamnoides* in China are average annual temperature, temperature seasonality, and precipitation in the coldest season. Collectively, these factors determine the formation and distribution patterns of suitable habitats.

In addition to climate factors, various other factors can influence the geographical distribution of plants. For instance, vegetation cover, topographic conditions, and soil characteristics may also play significant roles (Yang et al., 2018; Zhang et al., 2021; Churko et al., 2024). This study integrates topographic factors, soil properties, and human influences with climatic factors to provide a more accurate prediction of the potential distribution of *H. rhamnoides* in China over different time periods. This holistic approach deepens our understanding of the various factors shaping its distribution, thereby enhancing the model’s predictive accuracy. Previous studies have indicated that soil sand content can be a limiting factor for the distribution of *H. rhamnoides* in China. This research also examines the impact of soil sand content on its distribution (Zhang et al., 2024). Variations in slope gradients and orientations can indirectly influence soil moisture and light conditions, thereby affecting the distribution of *H. rhamnoides* in China (Jin et al., 2011). Therefore, when simulating the geographic distribution of species, it is crucial to consider a comprehensive array of environmental factors that influence their distribution. Additionally, this study primarily focuses on the potential distribution of *H. rhamnoides* under the current carbon emission scenario (SSP2-4.5), which may present certain limitations (Guo et al., 2021; Jiang et al., 2022).For instance, the potential distribution areas of *H. rhamnoides* under low-carbon emission pathways (SSP1-2.6), where environmental pressures are reduced, or under high-emission pathways (SSP5-8.5), where environmental conditions continue to deteriorate, are scenarios not addressed in this research.

## 5 Conclusions

This study employs an ensemble model to assess the potential distribution of Hippophae rhamnoides under current and future climate scenarios, focusing on environmental factors such as temperature, precipitation, and human activity impacts. The results reveal that the species’ suitable habitat area in China currently spans 2,128,900 km², primarily located in the Yellow River basin, with significant potential shifts in the coming decades. By 2050, 2070, and 2090, suitable habitats are expected to expand in the west while contracting in the east, with a westward shift in the centroid of these areas. These findings highlight the significant impact of climate change on the distribution of *H. rhamnoides* and emphasize the importance of targeted conservation efforts, especially in the western regions. The results provide a critical foundation for the sustainable management and conservation of *H. rhamnoides*, contributing not only to its protection but also offering valuable insights for the conservation of other key plant species. This study underscores the importance of incorporating climate projections into conservation strategies to safeguard biodiversity in the face of ongoing environmental changes. These findings highlight the significant impact of climate change on the distribution of *H. rhamnoides* and emphasize the importance of targeted conservation efforts, especially in the western regions. The results provide a critical foundation for the sustainable management and conservation of *H. rhamnoides*, contributing not only to its protection but also offering valuable insights for the conservation of other key plant species. This study underscores the importance of incorporating climate projections into conservation strategies to safeguard biodiversity in the face of ongoing environmental changes.

## Data Availability

The original contributions presented in the study are included in the article/supplementary material. Further inquiries can be directed to the corresponding author.

## References

[B1] Albuquerque J. P. Silva C. Ramos R. S. Zanuncio J. C. Castellani M. A. (2023). *Diaspis echinocacti (hemiptera: diaspididae*) on cactus pear cladodes: biological aspects at different temperatures. Braz. J. Biol. 83, e274016. doi: 10.1590/1519-6984.274016 37610947

[B2] Arora R. Bharti V. K. Dey S. (2024). Unlocking the potential of trans-himalayan high-altitude seabuckthorn (*Hippophae rhamnoides*) plants in the green synthesis of silver nanoparticles against drug-resistant foodborne pathogens: a step towards sustainable food safety goals. Nano 19, 2450024. doi: 10.1142/S1793292024500243

[B3] Balke I. Zeltina V. Zrelovs N. Kalnciema I. Resevica G. Ludviga R.. (2022). Identification and full genome analysis of the first putative virus of sea buckthorn (*Hippophae rhamnoides* l.). Microorganisms 10, 1933. doi: 10.3390/microorganisms10101933 PMC9609781 36296209

[B4] Bi Y. Xu J. Li Q. Guisan A. Thuiller W. Zimmermann N. E.. (2013). BioMod integrated multiple models to study the spatial distribution of species: A case study of the potential distribution of *hemlock* in China. J. Plant Taxonomy Resour. 35, 647–655.

[B5] Cai Y. Aihara T. Araki K. Sarmah R. Tsumura Y. Hirota M. (2024). Response of stomatal density and size in *betula ermanii* to contrasting climate conditions: the contributions of genetic and environmental factors. Ecol. Evol. 14, e11349. doi: 10.1002/ece3.11349 38895564 PMC11184283

[B6] Cheng Z. Chen Y. Yuan H. (2024). Molecular mechanisms of seed germination in *hippophae rhamnoides* l. Based on transcriptomics. Res. Cold Arid Regions 16, 310–322.

[B7] Churko E. E. Nhamo L. Chitakira M. (2024). Multispectral remote sensing approach of predicting the potential distribution and evaluating the current spread of water hyacinth (*Eichhornia crassipes*). Sust Wat Resour Man 10, 35. doi: 10.1007/s40899-023-01019-6

[B8] Duyar A. Demir M. A. Kabalak M. (2025). Prediction of current and future distributions of *chalcophora detrita* (coleoptera: buprestidae) under climate change scenarios. Ecol. Evol. 15, e70693. doi: 10.1002/ece3.70693 39830704 PMC11739133

[B9] Fu C. Wang Z. Peng Y. Zhuo Z. (2024). The potential distribution prediction of the forestry pest *Cyrtotrachelus buqueti (guer)* based on the maxent model across China. Forests 15, 1049. doi: 10.3390/f15061049

[B10] Gao H. Wei X. Peng Y. Zhuo Z. (2024). Predicting the impact of climate change on the future distribution of Paederus fuscipes curtis 1826, in China based on the maxent model. Insects 15, 437. doi: 10.3390/insects15060437 38921152 PMC11203407

[B11] Grimmett L. Whitsed R. Horta A. (2020). Presence-only species distribution models are sensitive to sample prevalence: evaluating models using spatial prediction stability and accuracy metrics. Ecol. Model. 431, 109194. doi: 10.1016/j.ecolmodel.2020.109194

[B12] Guo M. Ma S. Wang L. Lin C. (2021). Impacts of future climate change and different management scenarios on water-related ecosystem services: a case study in the jianghuai ecological economic zone, China. Ecol. Indic 127, 107732. doi: 10.1016/j.ecolind.2021.107732

[B13] Gupta R. Deswal R. (2012). Low temperature stress modulated secretome analysis and purification of antifreeze protein from *Hippophae rhamnoides*, a himalayan wonder plant. J. Proteome Res. 11, 2684–2696. doi: 10.1021/pr200944z 22486727

[B14] Hu J. Wang B. Bai L. Li Y. Zhang X. Liu J.. (2024). Quantifying the contribution of shrub roots to soil mechanical reinforcement using in *situ* shearing and assessing model reliability in coal mine subsidence areas, China. Catena 246, 108459. doi: 10.1016/j.catena.2024.108459

[B15] Huang L. Li S. Huang W. Xiang H. Jin J. Oskolski A. A. (2023). Glacial expansion of cold-tolerant species in low latitudes: megafossil evidence and species distribution modelling. Natl. Sci. Rev. 10, nwad38. doi: 10.1093/nsr/nwad038 PMC10029839 36960221

[B16] IUCN (2017). Hippophae rhamnoides The IUCN Red List of Threatened Species. Available online at: https://www.iucnredlist.org/species/55686342/119996497 (Accessed February 25, 2025).

[B17] Jiang J. Jin L. Huang L. Wang W. (2022). The future climate under different co2 emission scenarios significantly influences the potential distribution of *Achnatherum inebrians* in China. Sustainability-Basel 14, 4806. doi: 10.3390/su14084806

[B18] Jin T. Fu B. Liu G. Hu C. Su C. Liu Y. (2011). Diurnal photosynthetic variation of *Seabuckthorn* at different slopes and its main environmental factors. Acta Ecologica Sin. 31, 1783–1793.

[B19] Jin Z. Li Y. Wen X. Gu Y. Jin S. Guo H. (2006). Effects of drought on fruit yield in seabuckthorn plantations in China. Int. Seabuckthorn Res. Dev. 02), 31–36.

[B20] Jinga P. Liao Z. Nobis M. P. (2021). Species distribution modeling that overlooks intraspecific variation is inadequate for proper conservation of marula *(Sclerocarya birrea, anacardiaceae)* . Glob Ecol. Conserv. 32, e1908. doi: 10.1016/j.gecco.2021.e01908

[B21] Kearney M. R. Wintle B. A. Porter W. P. (2010). Correlative and mechanistic models of species distribution provide congruent forecasts under climate change. Conserv. Lett. 3, 203–213. doi: 10.1111/j.1755-263X.2010.00097.x

[B22] Kukina T. P. Shcherbakov D. N. Gensh K. V. Panteleyeva N. V. Tulysheva Y. A. Sal Nikova O. I.. (2020). Bioactive components in methyl tert-butyl ether extract of sea buckthorn (*Hippophae rhamnoides* l.) Green waste. Russ J. Bioorg Chem. 46, 1372–1377. doi: 10.1134/S1068162020070067

[B23] Lee D. E. Park K. H. Hong J. H. Kim S. H. Park K. M. Kim K. H. (2023). Anti-osteoporosis effects of triterpenoids from the fruit of sea buckthorn (*Hippophae rhamnoides*) through the promotion of osteoblast differentiation in mesenchymal stem cells, c3h10t1/2. Arch. Pharm. Res. 46, 771–781. doi: 10.1007/s12272-023-01468-9 37751030

[B24] Li G. Zhao Y. Tang D. Nie X. (1999). Relationship between the growth process and hydrothermal conditions of *Seabuckthorn* in China in the Mu Us Sandy Land. J. Northwest Forestry Univ. 01), 12–17.

[B25] Liu B. Deng X. Liu Z. Wei X. Zhang H. Xu D.. (2024). Predicted spatial patterns of suitable habitats for *troides aeacus* under different climate scenarios. Insects 15, 901. doi: 10.3390/insects15110901 39590500 PMC11594763

[B26] Lv Z. Yuan W. Zhang B. Xing G. (2021). Mass distribution of main active ingredients in *Sea buckthorn* fruit. J. Beijing Forestry Univ. 43, 144–152.

[B27] Ma Q. He N. Huang H. Fu X. Zhang Z. Shu J.. (2023). *Hippophae rhamnoides* l.: A comprehensive review on the botany, traditional uses, phytonutrients, health benefits, quality markers, and applications. J. Agr Food Chem. 71, 4769–4788. doi: 10.1021/acs.jafc.2c06916 36930583

[B28] Mihal M. Roychoudhury S. Sirotkin A. V. Kolesarova A. (2023). *Sea buckthorn*, its bioactive constituents, and mechanism of action: potential application in female reproduction. Front. Endocrinol. 14. doi: 10.3389/fendo.2023.1244300 PMC10662087 38027169

[B29] Petrie R. Denvil S. Ames S. Levavasseur G. Fiore S. Allen C.. (2021). Coordinating an operational data distribution network for cmip6 data. Geosci. Model. Dev. 14, 629–644. doi: 10.5194/gmd-14-629-2021

[B30] Qiu H. Han H. Cheng X. Kang F. (2025). Understanding sustainability of woody species suitability zones on the loess plateau for optimal creation zone selection in response to future climate change. J. Environ. Manage. 375, 124239. doi: 10.1016/j.jenvman.2025.124239 39874697

[B31] Su Y. Li S. Jiang H. Duan B. Liu M. Zhang Y. (2023). Sex-specific physiological and growth responses to elevated temperature and co2 concentration in chinese seabuckthorn (*Hippophae rhamnoides subsp. Sinensis rousi*). Acta Physiol. Plant 45, 53. doi: 10.1007/s11738-023-03520-z

[B32] Thuiller W. Lafourcade B. Engler R. Araújo M. B. (2009). Biomod – a platform for ensemble forecasting of species distributions. Ecography 32, 369–373. doi: 10.1111/j.1600-0587.2008.05742.x

[B33] Wang W. J. He H. S. Thompson F. R. Spetich M. A. Fraser J. S. (2018). Effects of species biological traits and environmental heterogeneity on simulated tree species distribution shifts under climate change. Sci. Total Environ. 634, 1214–1221. doi: 10.1016/j.scitotenv.2018.03.353 29710627

[B34] Wang H. Liu H. Cao G. Ma Z. Li Y. Zhang F.. (2020). Alpine grassland plants grow earlier and faster but biomass remains unchanged over 35 years of climate change. Ecol. Lett. 23, 701–710. doi: 10.1111/ele.13474 32052555 PMC7154776

[B35] Wang Y. Liu H. Xu J. Yu S. Huang Y. Zhang Y.. (2023). Prediction of suitable planting areas of *Rubia cordifolia* in China based on a species distribution model and analysis of specific secondary metabolites. Ind. Crop Prod 206, 117651. doi: 10.1016/j.indcrop.2023.117651

[B36] Wang F. Yuan X. Sun Y. Liu Y. (2024). Species distribution modeling based on maxent to inform biodiversity conservation in the central urban area of chongqing municipality. Ecol. Indic 158, 111491. doi: 10.1016/j.ecolind.2023.111491

[B37] Wen X. Zhao G. Cheng X. Chang G. Dong X. Lin X. (2022). Prediction of the potential distribution pattern of the great gerbil (*Rhombomys opimus*) under climate change based on ensemble modelling. Pest Manag Sci. 78, 3128–3134. doi: 10.1002/ps.6939 35442553

[B38] Wu J. Wei X. Wang Z. Peng Y. Liu B. Zhuo Z. (2024). Mapping the distribution of *Curculio davidi fairmaire* 1878 under climate change via geographical data and the maxent model (cmip6). Insects 15, 583. doi: 10.3390/insects15080583 39194788 PMC11354663

[B39] Xia C. Gao A. X. Dong T. T. Tsim K. W. (2023). Flavonoids from *seabuckthorn* (*Hippophae rhamnoides* l.) Mimic neurotrophic functions in inducing neurite outgrowth in cultured neurons: signaling via pi3k/akt and erk pathways. Phytomedicine 115, 154832. doi: 10.1016/j.phymed.2023.154832 37121059

[B40] Xian X. Zhao H. Wang R. Huang H. Chen B. Zhang G.. (2023). Climate change has increased the global threats posed by three ragweeds (*Ambrosia l.*) In the anthropocene. Sci. Total Environ. 859, 160252. doi: 10.1016/j.scitotenv.2022.160252 36427731

[B41] Xie A. Wang Y. Tian N. Xie X. Xi S. Uhl D. (2024). New occurrence of the late jurassic *Xenoxylon* wood in the sichuan basin, southern China: wood anatomy, and paleobiodiversity implications. Palz 98, 5–15. doi: 10.1007/s12542-023-00671-9

[B42] Yang W. Wang Y. Webb A. A. Li Z. Tian X. Han Z.. (2018). Influence of climatic and geographic factors on the spatial distribution of *Qinghai* sp*ruce* forests in the dryland qilian mountains of northwest China. Sci. Total Environ. 612, 1007–1017. doi: 10.1016/j.scitotenv.2017.08.180 28892842

[B43] Zhang C. He L. Dong T. Deng D. Liu J. (2024). Responses of soil moisture and vegetation carrying capacity to climate change in different sandy land types of Ruoergai based on Biome-BGC model. Chin. J. Ecol. 43, 1833–1840. doi: 10.13292/j.1000-4890.202406.007

[B44] Zhang X. Jiang Y. Bi Y. Liu X. Li X. Sun T.. (2022). Analysis of potential suitable distribution areas of chinese *hippophae rhamnoides* based on the maxent model. J. Ecol. 42, 1420–1428.

[B45] Zhang S. Liu X. Li R. Wang X. Cheng J. Yang Q.. (2021). Ahp-gis and maxent for delineation of potential distribution of *Arabica coffee* plantation under future climate in yunnan, China. Ecol. Indic 132, 108339. doi: 10.1016/j.ecolind.2021.108339

[B46] Zhang Y. Liu Y. Qin H. Meng Q. (2019). Spatial migration prediction of the suitable distribution area of *Shanxi winged fruit oil tree* under the condition of climate change. J. Appl. Ecol. 30, 496–502. doi: 10.13287/j.1001-9332.201902.040 30915801

[B47] Zhao G. Tian S. Jiang E. Jing Y. Chen R. Wang X.. (2024). Coordination analysis of flood-sediment transportation, eco-environment, and socio-economy coupling in the governance of the yellow river basin system. Sci. Rep-Uk 14, 8090. doi: 10.1038/s41598-024-58759-4 PMC10998862 38582920

[B48] Zhao R. Wang S. Chen S. (2024). Predicting the potential habitat suitability of *Saussurea* species in China under future climate scenarios using the optimized maximum entropy (maxent) model. J. Clean Prod 474, 143552. doi: 10.1016/j.jclepro.2024.143552

[B49] Zheng M. Chen H. Li D. Luo Y. Mo J. (2020). Substrate stoichiometry determines nitrogen fixation throughout succession in southern chinese forests. Ecol. Lett. 23, 336–347. doi: 10.1111/ele.13437 31802606

[B50] Zhou J. Jiang T. Wang Y. Su B. Tao H. Qin J.. (2020). Spatiotemporal variations of aridity index over the belt and road region under the 1.5°c and 2.0°c warming scenarios. J. Geogr. Sci. 30, 37–52. doi: 10.1007/s11442-020-1713-z

